# Academic Teams and Commercialization in the Life Sciences

**DOI:** 10.3389/frma.2021.733073

**Published:** 2021-09-24

**Authors:** Paige Clayton, Maryann Feldman

**Affiliations:** ^1^ Georgia Institute of Technology, Atlanta, GA, United States; ^2^ Department of Public Policy, University of North Carolina at Chapel Hill, Chapel Hill, NC, United States

**Keywords:** entrepreneurial teams, academic entrepreneurship, technology commercialization, co-founders, life sciences

## Abstract

We review the literature on entrepreneurial team formation with a focus on data to study academic teams and summarize our empirical work on the life sciences industry. We consider how academics form teams to start new companies and the implications of various configurations on firm behavior with regards to patenting, survival and firm growth. We present several empirical challenges facing research on academic teams and conclude with suggestions for future research.

## Teams and Academic Entrepreneurship

The Covid-19 vaccine provides an example of the way that scientific teams work together to commercialize academic discoveries. Beginning with research conducted in academic labs that focused on shifting of the vaccine mechanism to RNA, start-up firms such as Moderna and BioNTech moved the technology forward. Finally, through partnerships with large pharmaceutical firms such as Pfizer, AstraZeneca and Johnson and Johnson, vaccines were mass produced and distributed (Loftus, 2021). The introduction of the vaccines occurred in record time in response to the global pandemic through the dedicated efforts of cross-organizational teams. However, this response would have only been possible with efforts that extended back decades as start-up firms were created to advance academic discoveries and experiment with new technologies that were deemed too risky and early stage for big pharma.

Science-based start-ups exist at the intersection of academic discovery and commerce. The logic of teams is that the sum of the knowledge of the group members is greater than the sum of their individual contributions. Nowhere are team relationships more important than in the commercialization of academic discoveries, which is an essential step in realizing the social value of science. Scientific start-ups provide a means to take basic discoveries outside of academic labs and advance technology towards its commercial potential. This process requires a blending of skills: there is a need to be fully immersed in the science while simultaneously understanding the specialized vagaries of protecting intellectual property, market analysis and the banal tasks of meeting payroll. These tasks are so varied that the majority of science-based start-ups are formed by teams rather than single entrepreneurs. Academic scientists have specialized expertise about breakthrough science but lack the business acumen important to start companies to advance the science and make a product introduction to the commercial market (Clarysse and Moray, 2004). They often need to rely on external networks and CEOs to move from an initial firm creation to development and growth phase (Hayter, 2016; Huynh et al., 2017). Teams that blend these types of expertise are required to move science out of the lab to benefit the public.

Academic teams may be traced through scientific publication co-authorship, patent co-inventorship, or even co-principal investigator listings on federally sponsored research as these data are more readily available. Tracing founding teams and discerning the background of the entrepreneurs requires new methods of uncovering details about start-up firms and the career paths of their founders. This presents a data challenge as start-up companies may fail before showing up on traditional firm registries. Companies often change their names, or add or drop founders, making tracing their progress difficult. In addition, there are many teams that compete in business plan competitions or other entrepreneurial support activities without ever formally incorporating to move from a project to a bona fide company. There are many important questions that reliable data on founders and start-ups could uncover, with information on team founding patterns being one important subject. Our contribution is, first, to draw attention to innovative methods of collecting data on entrepreneurial firms and entrepreneurs that will allow advance of the study of academic entrepreneurial teams. We present a call to action to continue this data pursuit in other regions, sectors, and contexts. Our next contribution is to present a series of specific suggestions for future research on entrepreneurial teams based on our work studying North Carolina’s Research Triangle Region.

In this paper, we summarize some of the data sources used in the literature to examine academic teams in commercial activity, with a specific focus on startup firms. We then turn to a description of our own work that uses a unique database to study and contextualize the career histories of life science entrepreneurs in the Research Triangle Region. We describe the data and then report on the results on our research. We conclude with policy implications and suggestions for further research.

## Data to Study Academic Entrepreneurial Teams

Data to study academic teams involved in commercialization is not generally available and requires original data collection. Bercovitz and Feldman (2008), Bercovitz and Feldman (2011) have written papers that rely on administrative data collected from the technology transfer offices of universities. Access to these data require building relationships with university research officials and signing memorandum of understanding with the university offices. Generally, such data is collected for reasons other than supporting academic study and there is a need for significant data cleaning and curation of variables from other university offices. These studies are typically limited to one or a few universities. Results can be a comprehensive view of the process from research funding to publications to the filing of invention reports, which then leads to intellectual property (IP) protection. Bercovitz and Feldman (2011) examine the influence of characteristics of inventing teams on innovation outcomes and rely on invention reports, as a precursor to patent filings. In their 2008 paper, Bercovitz and Feldman explore how organizational work environments within university departments condition commercialization outcomes. While these papers provide a necessary, in-depth view of academic teams, larger scale studies are required for a more generalizable view.

Empirical research using national patent or publication databases to look at academic teams helps achieve greater generalizability with the trade-off of a less in-depth view. The advantage is a large number of observations. Scientific publications provide author names and institutional affiliations (Glänzel and Schubert, 2004; Giunta et al., 2016). Patent data provide the names, location and affiliations of inventors (Balconi et al., 2004; Crescenzi et al., 2017). These affiliations allow mapping to the university. One limitation is inconsistency in the reporting of author’s names, such as the use of initials, rather than full names or the use of middle initials. Matching models based on co-authoring networking relationships and topics allow for probabilistic modeling (Li et al., 2014). Of course, name changes due to life events among women authors and inventors yield undercounts of their activity (Colyvas et al., 2012). New, sophisticated big data techniques allow the linking of publications and patents (Bikard and Marx, 2020).

Empirical research has also used online start-up directories as a starting point to compile data to study academic teams. Roche et al. (2020) scrape crunchbase for U.S. biomedicine start-ups and supplement the data with Internet searches for firm founders. They use this dataset to examine whether team composition, specifically whether teams are academic or non-academic, influences venture outcomes. They find academic teams are less likely to achieve liquidity events, but patent more often than non-academic teams and have no difference in funding outcomes. Roche and colleagues further distinguish student, professor, and “superstar professor” startups, finding firms founded by the latter category outperform the others in terms of liquidity and in line with non-academic firms.

Of specific interest in the commercialization of science is the licensing of IP, which moves a discovery out of the university and enables either large firms or entrepreneurial start-ups to utilize the technology. Ali and Gittleman (2016) study how the composition of inventing teams matters for the licensing of medical innovations. Their specific context is inventions at two world-class academic medical centers: the Massachusetts General Hospital and the Brigham and Women’s Hospital, both affiliated with Harvard Medical School. Examining licensing over 30 years, their results indicate that medical clinicians are more likely to have licensed inventions than PhD researchers. Team led by clinicians had increased rates of commercial licensing due to the emphasis of their training on translational research that would be of greater interest to industry.

Entrepreneurial startups are of more interest than licensing to large firms. There is evidence that licensing to large firms has declined in favor of a new pathway of large firms investing in and/or acquiring start-up firms. Many studies consider academic start-ups (Nikiforou et al., 2018 for a review). Once again, these studies tend to use university administrative data to collect the names of the firms and then match with other data sources. These studies examine firms who have licensed from universities even while we recognize that many academic entrepreneurs circumvent the bureaucracy of the university technology transfer offices. Of issue is the fact that members of the founding team may be from outside the university and full details of their career history would be missing.

In summary, while there has been interest in the topic of academic teams over the past 15–20 years, the inability to access adequate data has been the limiting factor. The availability of digital data and increased computation power permits the linking of different data sources to form a more comprehensive overview of academic teams and the commercialization of science. As universities across the U.S. continue to prioritize commercialization and spin-outs, better understanding academic founding teams will be important. In the next section we present our work to develop a novel database that allows the tracking of entrepreneurial teams and comparison of academic to non-academic teams.

## Academic Teams in Research Triangle, North Carolina

Academic entrepreneurship is viewed as an important economic development policy lever. Understanding the dynamics of start-up team formation and the subsequent effect on firm survival and employment provide a means to increase the economic performance of a region. Our work utilizes a unique database to study and contextualize the career histories of life science entrepreneurs in North Carolina’s Research Triangle region. These data enable us to capture and compare different prior employment experiences in order to better understand how the team composition impacts entrepreneurial firms and the surrounding region. The data on life science firms and their founders are drawn from the PLatform for Advancing Community Economies (PLACE): Research Triangle database, which collects information about the Research Triangle region’s entrepreneurial ecosystem from over 30 data sources (Feldman and Lowe, 2015)^1^. These data allow an investigation of the dynamics within an entrepreneurial ecosystem.

PLACE: Research Triangle is unique in the depth of information covered on start-up firms and their founders, as well as in the breadth of information contained on ecosystem members, such as support organizations, and how firms interact with them. Furthermore, the database follows start-ups over their life in order to track milestones. PLACE is longitudinal and takes an historical perspective on the dynamics of the ecosystems and firms that comprise it. The database was first compiled based on historical records of life sciences firms founded in the region. These lists were compiled, de-duplicated, and vetted by local industry leaders and entrepreneurial ecosystem champions. After the universe of start-ups was compiled, work commenced to gather information on who the founders were, and then on the founders’ work histories and educational backgrounds. Researchers also gathered data from local entrepreneurial support organizations and local and national funding sources to develop an understanding of firm support and growth. Database building required matching proprietary sources including National Establishment Time Series, CB Insights, crunchbase, and North Carolina Biotech Center data to publicly available sources such as the North Carolina Secretary of State records and National Institutes of Health (NIH) Small Business Innovation Research (SBIR) funding records. Data on founders was obtained through targeted Internet searches of career-based social media platforms, company websites, news articles, press releases, Securities and Exchange Commission filings, business journals, crunchbase, journal article affiliations of academic founders, and many other sources.

**FIGURE 1:**
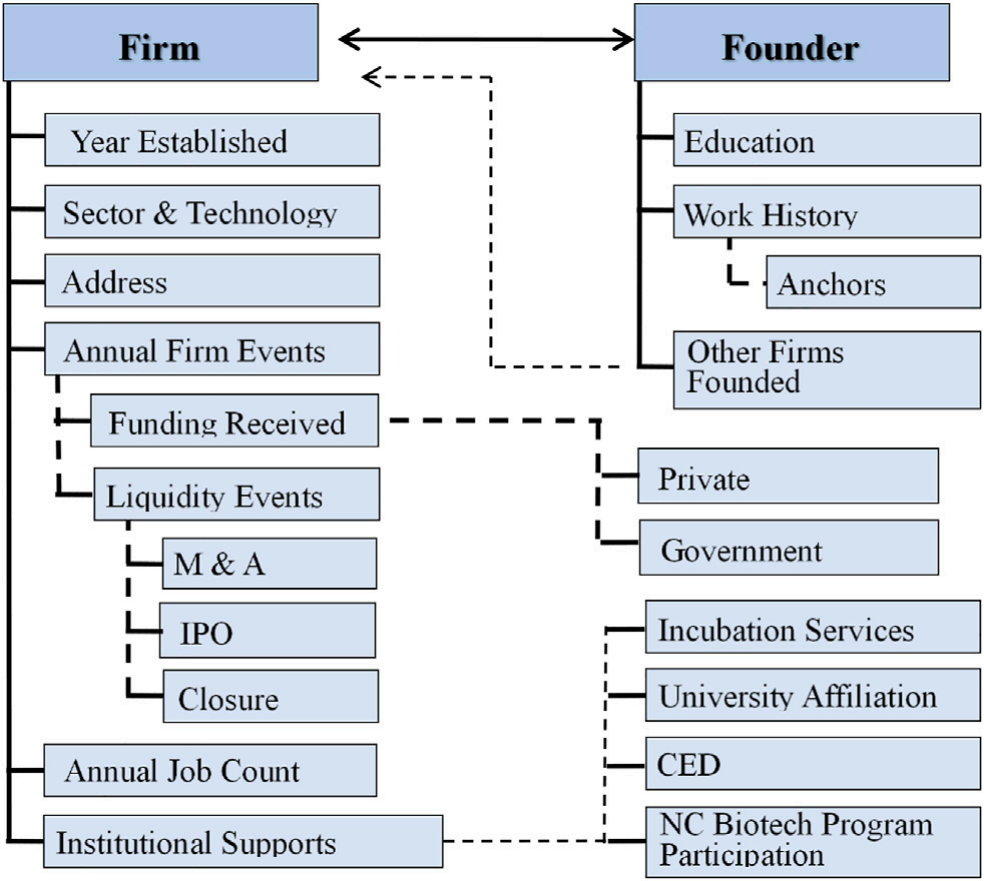
Place database MySQL relational structure. Source: Feldman and Lowe (2015).

This extensive data compilation effort required the work of over ten research assistants over several years. The result is a unique and rich relational database hosted on a MySQL platform that allows for easy data entry, reporting, and visualization. MySQL relational data structure is depicted in Figure 1. Investigating questions around entrepreneurial team formation and patterns are just one of many areas for which the data may be used.

**FIGURE 2:**
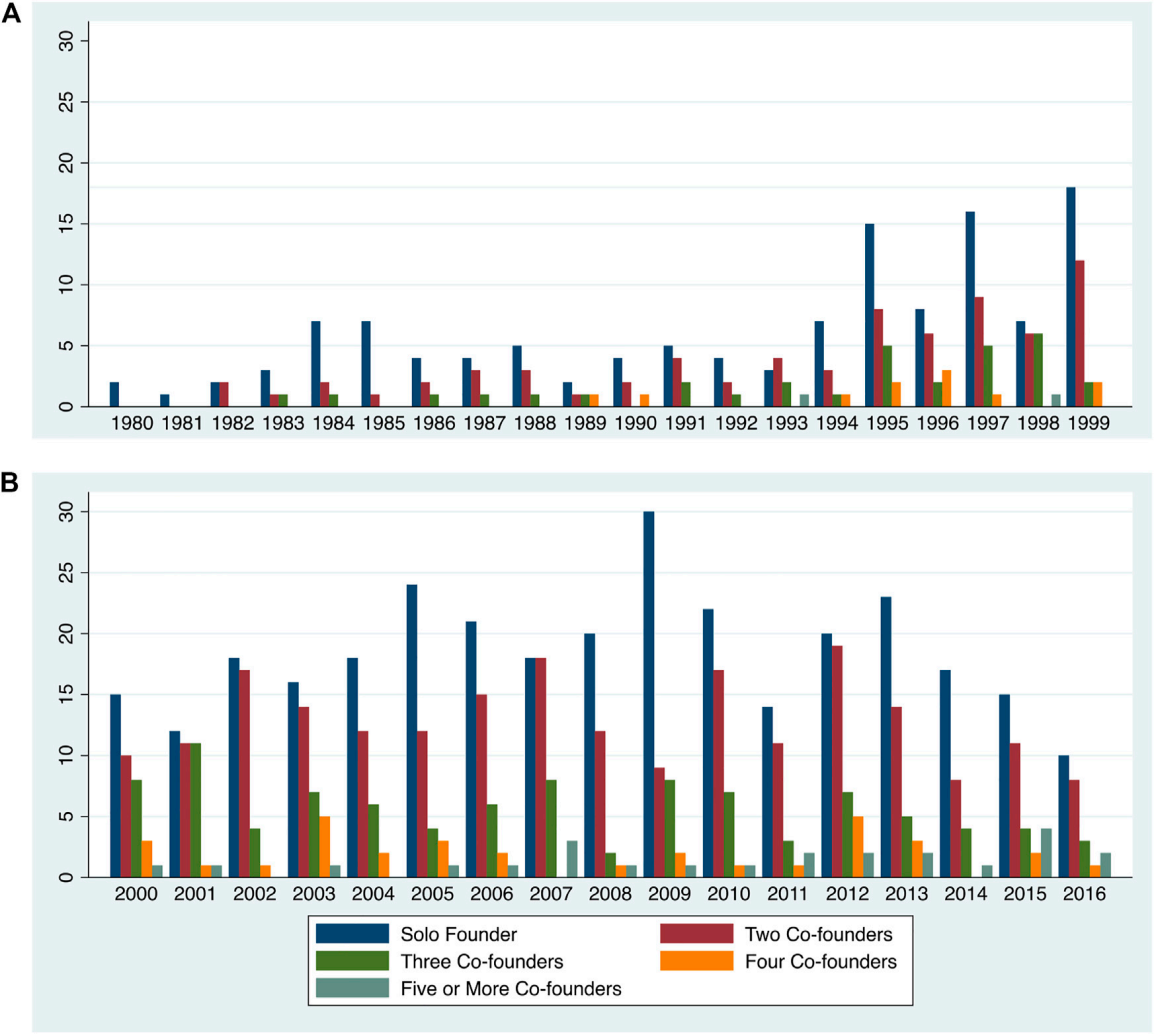
Entrepreneurial team size distribution, 1980–2016. Source: Author based on PLACE: Research Triangle database.dslb

The curated life sciences population in the PLACE: Research Triangle database currently contains information on the Universe of 925 life sciences firms founded from 1980 through 2016 in the Triangle. Of these, 52.3% of firms were founded by teams. The most common team size is two-person teams, comprising 31.24% of firms. Team sizes become larger over time, as shown in Figure 2.

Of the 52.3% of firms founded by teams, 47.4% have at least one academic cofounder from one of the region’s three research universities: Duke University, the University of North Carolina at Chapel Hill, or North Carolina State University. 11.3% of teams are composed entirely of academic co-founders. The composition of these teams also shows variation with time, as more academics appear on founding teams in more recent years. In other work (Clayton et al., 2019), we demonstrate that the Research Triangle’s life sciences industry has experience three development stages based on industry dynamics: a nascent stage, accelerated growth, and maturing growth (we explore this work further in a later section). When examining team composition across these industry stages we make the following observations. In the early, nascent industry stage (lasting from 1980–1997), 50% of teams had academic co-founders, while only 4.2% were composed entirely of academics. During the stage of accelerating, rapid entrepreneurial growth (1998–2009), we see a smaller percentage of firms founded with academic co-founders on their founding teams (41.3%), but a greater percentage of firms with only academic co-founders (9.3%). Finally, in the most recent stage of maturing growth (starting in 2010), we see the greatest extent of academic entrepreneurship: 57.4% of firms are founded with at least one academic co-founder and 18.24% have only academic start-up teams. One explanation for this change is the gradual legitimation of entrepreneurship as an activity of university faculty that has occurred over the past two decades. Universities have changed policies to allow faculty leave to pursue entrepreneurial ventures, a sign of the their recognition of the positive effects such activities may have for the university.^2^

These descriptive results from the PLACE: Research Triangle database illustrate the utility of developing new data sources for studying entrepreneurial phenomenon. In the next three sections we outline three of our studies which make use of this data and highlight their findings as they relate to academic founding teams.

## Founding Teams and Ecosystem Dynamics

Clayton et al. (2019) study relationships between the prior work experiences of entrepreneurial founding teams and their firms. Former studies suggest that the organizational logics, knowledge, and expectations that the founders develop through prior work impact the new firms’ organization, profitability, and success (Feldman et al., 2021). Breakaway firms, which are founded by entrepreneurs with experience in the same industry, drive the growth of innovative clusters (Jacobs, 1969). Entrepreneurs learn to set up new firms through experiences and know-how gained from their prior employment. In this way, prior work experience is viewed as a *de facto* apprenticeship. The skills that founding team members bring to their new firm shape which products and processes the newly founded firms’ exploit. These *de facto* apprenticeships also offer blueprints for how the teams of entrepreneurs organize and position their new firm within existing markets.

Existing scholarship tends to concentrate on two dominant career or apprenticeship tracks, often studied independent of the other: those associated with prior work at large corporations *or* those stemming from prominent academic institutions. In Clayton et al. (2019), we instead provide a more holistic, localized view of pre-entry experiences’ effect on firm dynamics by distinguishing 1) local academic, 2) local large pharmaceutical, 3) local branches of multinational corporations, and 4) local entrepreneurial experience.

For this study, we limit our sample to 460 entrepreneurial life science firms founded between 1980 and 2012. These firms are linked to 872 founders with known work experience. We study the outcomes of patenting, firm survival, likelihood of experiencing a merger or acquisition, and employment; we also assess the association between experience types and the levels of private and public funding received. We include founding team size, age of firms, and a dummy variable for human therapeutics firms as variables. Table 1 displays information about founding teams by local pre-entry experience type.

**TABLE 1:** Distribution of founder local work experience type.

Firms, with any founder work experience in category	Total number of firms, with experience type	Average number of founders per firm
Local academic	206	2.30
Local pharma	152	2.10
Multinational corp. local branch	76	2.28
Local entrepreneurial	198	2.14

*Source*: Adapted from Clayton et al. (2019).

*Notes*: The mean number of founders is 1.90 per firm. Sixty-five percent of founding teams have at least one co-founder with local prior work experience in these categories.

We find that among firms whose founders experienced local apprenticeships, the age of the firms, the number of founders, the level of private funding, and being in the human therapeutics sector positively correlate with start-ups’ employment level. Startups with founders who have academic experience tend to have smaller firms in terms of employment size. This accords with findings that academic entrepreneurs do not value growth as an outcome of their entrepreneurial efforts as much as non-academic entrepreneurs (Hayter, 2011). Work experience at local entrepreneurial life science firms is positively correlated with employment for all yearly snapshots. However, this finding does not hold when the model includes all 460 firms–it is only apparent in the apprenticeship sub-sample.

Although the firms founded by founders with experience at local entrepreneurial firms have a relatively positive employment level, the most consistent employment effects are more related to the founding team size, technology choice, and firm age than the prior work experience or pedigree of the founders. Firms with a greater number of co-founders tend to also employ the largest number of employees across both apprenticeship firms and all firm samples. This relationship persists through year nine employment. Other economic metrics in the analysis, such as patenting, show that academic-affiliated firms tend to introduce more new products and processes compared to non-academic-affiliated firms, so this type of contribution from academic-affiliated firms is greater than direct job creation.

Clayton et al. (2019) findings provide policy implications for regional decision-makers and practitioners. We acknowledge the need for further research on the impacts of academic entrepreneurship on local labor markets. Importantly for this paper, Clayton et al. (2019) demonstrates the importance of considering how founding team composition relates to firm growth outcomes. The paper’s contribution is to consider academic and non-academic work experience together, as many studies looking at academic teams often only study samples of academic teams.

## Teams and Entrepreneurial Apprenticeships

Just as academics learn to conduct research by working in their advisors’ labs, entrepreneurs learn how to set up new firms through experience gained from their prior employment (Aldrich, 1999; Zott and Amit, 2007; Sørensen and Phillips, 2011). A series of empirical studies link the performance of newly founded ventures to the entrepreneur’s prior employers in private sector firms (Burton et al., 2002; Dahl and Reichstein, 2007; Chatterji, 2009; Dencker et al., 2009; Ganco and Agarwal 2009; Semadeni and Cannella, 2011; Ferriani et al., 2012; ). Many firms are started by individuals after they worked for a prominent, well-established corporation within a region—a well-studied phenomena described as “spawning” (Klepper 2002). Prior work experience may be viewed as a *de facto* apprenticeship, shaping which how these entrepreneurs organize their new firms, which determines the firm’s ability to survive, attract resources and generate employment.

Another strain of literature examines entrepreneurship by academics seeking to commercialize scientific discoveries. Employees of a research university are more likely to have a scientific orientation and access to resources from technology transfer offices and business school initiatives to help them commercialize technological discoveries (Saxenian, 1996; Trippl and Todtling, 2007; Link et al., 2015). The literature has also featured star scientists–individuals of high academic achievement that form highly successful entrepreneurial firms (Zucker and Darby, 1996). Alternatively, those entrepreneurs with no prior work experience in these leading academic and prestigious corporate settings may exhibit the worst performance, or constrain the performance of teams they join.

Most new firms are founded by teams of individuals who combine various types of expertise (Franklin et al., 2001). Founding teams can draw from both corporate and academic experiences, thus blending different organizational experiences and apprenticeships. Still other entrepreneurial founders first gain experience as employees of local start-up firms, thus becoming part of a second or subsequent generation of entrepreneurial firms, positioning them with greater expertise about entrepreneurial opportunities.

Much less is known about the influence of prior employment on subsequent entrepreneurial choices regarding firm strategy, especially regarding size and growth trajectory—that is to say, whether an entrepreneurial founder seeks to support a large, fast-growing operation or rather seeks to maintain a slow but steady pace of organizational growth and keep their entrepreneurial venture small and nimble. In Donegan et al. (2019) we characterize these two trajectories by invoking the classic parable of the tortoise and the hare, where the fast-growing operation behaves as the hare and the steadier paced firms behave as the tortoise. Our title, “The tortoise, the hare, and the hybrid”, however, acknowledges alternative hybrid pathways may operate as well that blend these two trajectories. Tracing distinct founders’ prior work experiences to patterns of organizational growth and funding acquisition helps us to better situate entrepreneurial aspirations and decision-making. By differentiating the labor market effects of a concurrent set of entrepreneurial pathways, we can inform regional economic development strategy and give practitioners, who hope to leverage entrepreneurship for regional job creation, better insights for targeting public funding and policy support.

In Donegan et al. (2019), we argue that entrepreneurs’ prior employment experience will lead them to pursue certain entrepreneurial pathways in their local entrepreneurial ecosystem at the expense of other pathways. When multiple entrepreneurs work together to start a firm as a team, this pathways interact and further influence outcomes. This argument has based on a number of studies finding that corporate or academic founders tend to follow similar patterns in founding their firms but has not been examined in the context of an entrepreneurial ecosystem. For example Dencker et al. (2009), Roberts et al. (2011), Campbell et al. (2012) find that entrepreneurs with prior work at a large corporation often start businesses in fields closely related to their former employers. Donegan and Lowe (2020) and Wright et al. (2004) reveal that academic entrepreneurs often generate new products and ideas through laboratory and applied research; universities provide support and resources in networking, mentoring, and funding, which ultimately shapes the structure of those companies. Franklin et al. (2001) find that the difficulty to balance the academic job and the entrepreneurial work may impact the firm’s long-term performance. Hybrid teams can combine both experiences (Dahl and Sorenson, 2012).

This study examines 488 firms that were founded between 1990 and 2012 for which both founder employment history and yearly employment numbers for the firm are available. The 488 firms are linked to 924 founders that are categorized by prior employment types: academic employers, entrepreneurial life science firms, big pharmaceutical corporations, and hybrids of two or more types. We find that there is considerable career heterogeneity among entrepreneurial founders which relates to differential performance outcomes for firms. Results are provided in Table 2. The results indicate that firms whose growth patterns behave like the parable’s tortoise are more likely to have a merger or acquisition when they have big-pharma experienced founders, and are more likely to survive when they have academic founders. In contrast, the firms with second-generation entrepreneurial founders, which behave like the parable’s hare, do not show any significant results for their survival and exit outcomes. Firms associated with higher proportions of academic experience shows a higher survival rate and more success in raising public financing; big pharma firms are more likely to experience a merger or acquisition. Donegan et al. (2019) suggest that understanding the former experience of local entrepreneurs can provide information useful to support and reinforce policy consequences. Compared to big pharma, entrepreneurial, or any combination of experience, we find that academic experience is positively related to receiving public startup funding and academic apprenticeship founded firms are more likely to survive. This is positive news for academic start-ups and demonstrates how these teams may leverage existing strengths in research grantsmanship to acquire public funding. Our categorization of prior experience could be built out further to distinguish student entrepreneurs and to look in different regional and national contexts.

**TABLE 2:** Summary Results of Donegan et al. (2019).

	Public funding	Private funding	Patenting	Patent count	Survival	Merger and acquisition	Employment
Academic experience	Positive	Negative	Negative	Negative	More likely to survive	—	Positive (years 3, 6, 9)
Big pharma experience	Negative	—	Negative	Positive	—	Positive	Positive (years 3, 6, 9)
Entrepreneurial experience	Negative	Negative	Negative	Negative	—	—	Positive (all years)
Academic-pharma experience	—	Negative	Negative	—	—	—	Positive (last year)
Academic-pharma-ent experience	—	—	—	—	—	—	—
Entrepreneurial-pharma experience	Negative	Negative	Negative	Positive	—	—	—
Entrepreneurial-academic experience	—	Negative	—	—	—	—	—

Source: Replicated from Donegan et al. (2019).

## Defining Industry and Time in Regional Entrepreneurship Research

As illustrated in the earlier section “Academic Teams in Research Triangle, North Carolina”, the patterns of academic entrepreneurial teams change over time. In other research, we empirically define the timing of industry stage development in the Research Triangle and use these stages to examine how entrepreneurial finance matters differently over time for firm survival (Clayton et al., 2019). Using threshold regression on local industry dynamics (i.e., number of active startups) we identify two dates when the relationship between time and industry growth changed, which define three distinct industry life cycle stages. A nascent growth phase operated from 1980 until 1996, when a period of accelerated growth (larger slope) began and lasted until 2009. From 2010 to 2016 the slope decreased and the local industry entered a maturing growth stage. These stages and their growth rates correspond to a logistic s-shaped curve that is canonical to theory on industry development.

Using the universe of life sciences research and development focused firms founded from 1980 to 2016 we then examine whether three different institutional funding sources (state public, federal public, and private) exhibit different relationships to each other. Specifically, we want to know whether certain sources drive follow-on funding from other sources and if these relationships change depending on the stage of local industry development. Using regime switching analysis applied to a dynamic random effects probit model we demonstrate that the overall trends in finance relationships across the entire time period (1980–2016) mask considerable heterogeneity within different industry stages. For example, none of the three funding sources predicts future state funding, which usually invests early in smaller amounts, when looking at the overall time period. However, when examining by industry stage, private funding actually strongly predicts state funding during the maturing growth stage (stage 3). We reason that this is due to greater amounts of private seed funding becoming available in the region over time that would invest even sooner than public funding.

Finally, we examine whether these different funding sources help firms survive longer and whether funding sources’ relationship to survival also change depending on the stage of the industry life cycle. For this analysis we apply regime switching to a discrete event history analysis, using a complementary log-log model. We find no relationship between state, federal, or private funding and survival in the nascent stage, but both private and federal funding begin to relate to a decreased risk of failure in the acceleration stage. In the maturing growth stage, all three funding sources relate to a decreased risk of failure, with private funding having the strongest result statistically and economically. Considering these results alongside the funding relationships results illustrates that funding sources play evolving roles over time. For example, while state funding may play a signaling role early in the ecosystem’s development, its direct impact on firm survival only becomes apparent in the maturing growth stage.

While this paper is not specifically about teams, we present it here because this staged approach will be useful for future research examining local ecosystem patterns, including those highlighted above with regard to academic co-founding teams. For example, we showed the phase of maturing growth had the greatest share of academic involvement in founding teams. There is an opportunity to expand this stage-based research on ecosystems to many other aspects of local entrepreneurship and economic development. In the next section we discuss several avenues for advancing research on academic teams.

## Advancing This Methodology

Several methodological issues complicate research on academic entrepreneurial teams. We examine three here. First, methodologically, is the difficulty of ascertaining how much influence any one co-founder exerts on the startup process. While a founding team may have several members, it is not likely that they all exert the same influence on firm decisions and progression. For example, some co-founders may be more focused on moving the technology forward, while others focus on developing the business plan and seeking funding. Without survey data, it is difficult to assess. And while surveys are possible to conduct, they have well known downsides of being costly, time consuming, and suffering from response bias. These limitations require a tradeoff. Therefore, most research on academic teams has ignored this question.

Second is the issue of aggregation in quantitative analysis. While founders have individual characteristics, to study and compare teams across a sample of firms empirically there is a question of how to aggregate information in a way that provides the most in-depth and nuanced view of the team. For example, if trying to determine the level of human or social capital possessed by a team, there is a question of whether it is better to use an average of all team members, a maximum value, or even a minimum value. Researchers should report a range of values. Other variables of interest are founders’ age, educational attainment, gender, or race and ethnicity. Again, the issue of how to characterize a heterogeneous team along these lines becomes of issue. A common approach is to simplify by creating dummy variables for whether any co-founder possesses certain traits, but this simplification may be suboptimal for analysis.

Finally, is the question of how founding teams evolve over time, as the original founding team may not stay intact, and data tracking founders’ movements can be difficult to trace. For example, non-founding CEOs and other executives are often brought in at later development stages to help the firm scale. This is a question really about the length of time the founding team exerts power and whether the initial founding team composition has an impact that lasts for several time periods, even after they have moved on. Social media sources that provide job histories may be useful for answering this question, as they allow tracking of individual founders over time.

## Concluding Remarks and Suggestions for Future Research

This paper offers a review of literature on academic co-founding teams with a focus on data approaches. We provide a synthesis of our work building a dataset that can be used to meaningfully study founding teams. Our research highlights the utility of considering outcomes over a long time period and assessing changing patterns of team composition over time. By focusing on one place we hold other factor constant. This kind of research allows an examination of how industries evolve and indicates points where policy can intervene.

In sum, our papers show the variability in team composition in the life sciences–an industry that relies on academic discoveries as an input. Teams contain a blend of academics, individuals from large, incumbent life sciences firms, and individuals with prior start-up experience. We should note that the *de facto* apprenticeships that entrepreneurs experience in their prior employment imprint different logics of how firm formation occur, with certain teams pursuing quick growth, while others pursue more steady growth. We also show that team composition aligns with specific patenting and funding outcomes. While our research is limited to one region, our analysis is replicable in other places and the PLACE: Research Triangle database provides a useful template for future research. Furthermore, many of the issues in studying teams raised in this paper are cross-cutting and influence other industries as well outside the life sciences.

Our results have implications for universities that are encouraging academics to engage in starting up firms. Rather than forming a founding team with other academic founders, academics could be encouraged to team up with individuals with industry background. Including team members who have local experience working with similar startups positions a firm with very specific knowledge but also a network of local contacts. More work needs to be done to understand how teams are formed and how demographic diversity of team member contributes to firm performance.

Collecting better quality and real-time data to study teams also has implications for policy. By developing these data sources and building or leveraging local community partnerships, local policy makers and civic leaders can be provided with evidence about how to better support entrepreneurship and small, new firms. Our data has been analyzed and presented to officials in North Carolina which provides tangible proof of concept for the utility of our data methodology.

Future research should address not only the questions about how academic teams form, the boundaries around their effectiveness, and patterns in how their teams develop, but should also address the methodological issues that make studying teams difficult. Teams are an aggregation of individuals, whose actions together accomplish more than any individual alone. But it is precisely this emergent property that is difficult to capture empirically. Future research on academic teams should also consider supplemented the in-depth quantitative data that computing advances allow us to capture with qualitative data based on interviews that allow rich contextual details on entrepreneurial academic teams. Such interviews allow researchers to better understand motivations, conflicts and trust within teams, and support networks which are not readily ascertained by quantitative sources.

## Data Availability

The datasets presented in this article are not readily available because Data is proprietary. Requests to access the datasets should be directed to https://maryannfeldman.web.unc.edu/research-on-research-triangle/.

## Footnotes

1 More about PLACE at this link: https://maryannfeldman.web.unc.edu/research-on-research-triangle/

2 An additional descriptive we examined on founding teams was whether there were patterns of serial founders moving to larger or smaller teams in their successive entrepreneurial endeavors, but we found no patterns of this kind.
